# Hedging, ambiguity, and the rejection of misinformation: evidence from Chinese readers

**DOI:** 10.3389/fpsyg.2026.1880060

**Published:** 2026-07-27

**Authors:** Ruihong Li, Cun Fu

**Affiliations:** School of Foreign Languages and Cultures, Chongqing University, Chongqing, China

**Keywords:** ambiguity cues, hedging, misinformation evaluation, misinformation rejection, numerical ambiguity, reaction time, sharing intention, source ambiguity

## Abstract

Misinformation poses a challenge not only because false claims circulate widely, but also because readers must make judgments about whether such claims are credible and whether they should be shared. While hedging is frequently observed in misinformation, its effects on audience evaluation remain insufficiently differentiated, particularly outside Western linguistic settings. This study examines whether two ambiguity-based hedging cues, source ambiguity and numerical ambiguity, shape readers' responses to Chinese misinformation in distinct ways. A total of 182 participants evaluated a set of Chinese misinformation claims that had previously been debunked by authoritative fact-checking sources. The statements were presented in one of four versions generated through the presence or absence of source ambiguity and numerical ambiguity. Participants' rejection of debunked misinformation claims, sharing intentions, and reaction times were analyzed using mixed-effects models. The results showed that both source ambiguity and numerical ambiguity were associated with greater rejection of debunked misinformation claims. Neither cue directly predicted sharing intention; however, greater rejection of debunked misinformation claims was associated with lower willingness to share it. Numerical ambiguity, but not source ambiguity, was additionally associated with shorter reaction times. These findings suggest that ambiguity-based hedging cues may function as cues of unreliability rather than persuasive devices, highlighting the importance of source traceability and numerical specificity in misinformation literacy interventions.

## 1 Introduction

In digital communication environments, readers often encounter questionable claims before external verification is available. Misinformation, understood here as false or misleading information that may circulate regardless of intent, has therefore become a major concern in domains such as politics, health, and social issues (El Mikati et al., 2023). Its impact depends not only on how widely false claims spread, but also on how readers evaluate their credibility and decide whether they should be shared. Beyond individual misunderstanding, misinformation may also produce broader social harms, including the distortion of public opinion, the amplification of social anxiety, and the erosion of trust in institutions (Muhammed and Mathew, 2022). Accordingly, a growing body of research has examined the spread of misinformation (e.g., Vosoughi et al., 2018), the psychological processes underlying credibility evaluation and truth discernment (e.g., Pennycook and Rand, 2019, 2021; Luo et al., 2022), and strategies for correcting or mitigating its influence (e.g., Lewandowsky et al., 2012; Pennycook and Rand, 2021).

Most empirical and theoretical research on misinformation has focused on the psychological and social factors that shape its acceptance and spread, whereas far fewer have examined the linguistic and discursive features through which misinformation is constructed and communicated (e.g., Rashkin et al., 2017; Zhao et al., 2022; Zhao and Fu, 2024; Fu et al., 2024; Lutz et al., 2024). Yet such features are central to how misinformation is formulated and made interpretable to audiences. For example, Zhao et al. (2022) showed that content characteristics such as length, place, time, emotion, and calls for action were associated with the veracity of COVID-19 rumors, suggesting that message-level features may play a meaningful role in how readers evaluate questionable claims.

One important class of message-level cues involves ambiguity in evidential framing. In linguistic and pragmatic research, such expressions are often discussed as forms of hedging, because they reduce explicit commitment to propositional content and introduce uncertainty, tentativeness, or vagueness (Lakoff, 1972; Hyland, 1998). Common examples include reduced source identifiability, such as anonymous or weakly identifiable source attributions (e.g., Pjesivac and Rui, 2014), modal expressions (e.g., Hyland, 1998), and vague quantifiers (e.g., Solt, 2011). In misinformation contexts, such expressions may increase the apparent plausibility of claims while simultaneously reducing their verifiability (Rashkin et al., 2017). However, the psychological function of such cues is not straightforward. Ambiguity -related hedging cues may make a claim appear more cautious and therefore more plausible, but they may also alert readers to weak evidential support and reduce perceived reliability.

It remains unclear whether ambiguous language in misinformation functions primarily as a persuasive device or as a cue of unreliability. On one hand, deceptive messages can invoke or construct expertise and objectivity cues to legitimize questionable content (van Eck et al., 2026), and hedging may enhance source trustworthiness when it signals caution, transparency, or scientific uncertainty in science news contexts (Jensen, 2008). For instance, Zeng et al. (2023) found that uncertainty introduced through hedged language increased perceived credibility and news-sharing likelihood. On the other hand, hedging may also function as a cue of weak evidence or unreliability, thereby reducing trust. For example, Mitra et al. (2017) found that hedging expressions were associated with lower perceived credibility. Similarly, van der Bles et al. (2020) found that verbal communication of uncertainty around numerical information increased perceived uncertainty and reduced trust in the number, although effects on trust in the source were small or inconsistent. More broadly, Stavrova et al. (2024) and Orr et al. (2026) suggest that the effects of uncertainty and hedging are contingent on the form of expression, the evaluative outcome, and the communicative context. Taken together, these findings indicate that the effects of hedging are not uniform.

One possible reason for these mixed findings is that ambiguous language in misinformation has often been treated as a relatively broad or undifferentiated feature. In practice, however, ambiguity may arise from different aspects of a claim's evidential framing. Some expressions obscure where the information comes from, whereas others obscure the precision of quantitative information. Although previous studies have shown that uncertainty-related linguistic cues and source-related cues can influence perceived credibility (e.g., Rashkin et al., 2017; Mitra et al., 2017; van der Bles et al., 2020; Zeng et al., 2023; Stavrova et al., 2024), most have not explicitly distinguished between ambiguity in source attribution and ambiguity in numerical expression.

This issue may be usefully understood through the lens of *epistemic vigilance theory* (Sperber et al., 2010), which proposes that receivers monitor communicated information for cues bearing on source reliability and content credibility. From this perspective, ambiguity-based hedging may be relevant to readers' epistemic vigilance because it affects how evidential support is presented. The present study therefore focuses on two theoretically meaningful forms of ambiguity-based hedging: source ambiguity and numerical ambiguity. These two forms of ambiguity correspond to two types of cues that readers may use when evaluating communicated information: source reliability and evidential precision. In this study, source ambiguity refers to vague or untraceable attributions such as it is reported that, where the source of information is not clearly identifiable and such expressions may alter perceptions of speaker accountability and message credibility (e.g., Mahr and Csibra, 2021; Pjesivac and Rui, 2014). Numerical ambiguity refers to the use of imprecise verbal expressions to communicate quantitative or probabilistic information, which may affect judgment by reducing precision in the information conveyed (e.g., Budescu and Wallsten, 1995; Collins and Mandel, 2019).

Guided by *epistemic vigilance theory*, we therefore examine whether and how source ambiguity and numerical ambiguity, two ambiguity-based hedging cues that correspond to different aspects of evidential framing, are associated with readers' misinformation rejection, sharing intentions, and reaction times in Chinese misinformation discourse. Because their relative effects remain unclear, the comparison between these two ambiguity types is treated as exploratory. In addition to capturing evaluative outcomes, measures such as veracity judgments and reaction times may also provide useful evidence about how misinformation is processed (Rapp, 2008; Wertgen and Richter, 2020). For example, Rapp (2008) showed that participants took longer to read inaccurate than accurate information, even after sentence length was controlled.

Additionally, because user sharing is a key pathway through which misinformation spreads online (Lazer et al., 2018; Vosoughi et al., 2018), we conducted exploratory analyses of sharing intention. Prior research suggests that, under natural conditions, people's sharing decisions do not always closely follow their veracity judgments (e.g., Pennycook and Rand, 2021; Pennycook et al., 2021). For example, Pennycook and Rand (2021) argued that there is often a substantial disconnect between what people believe and what they choose to share on social media, partly because accuracy is not always salient at the moment of sharing. Therefore, we examined the extent to which readers' veracity judgments were associated with their subsequent sharing intentions.

Accordingly, the current study examined whether source ambiguity and numerical ambiguity shape Chinese readers' responses to misinformation, including their rejection of debunked misinformation claims, their willingness to share it, and their reaction times during misinformation rejection. It further examined whether misinformation rejection was associated with sharing intention.

## 2 Method

### 2.1 Research design and participants

This study employed a 2 (source ambiguity: specific vs. vague) × 2 (numerical ambiguity: precise vs. imprecise) between-subjects design, which resulted in four experimental conditions: low ambiguity (specific source + precise number), source-only ambiguity (vague source + precise number), numerical-only ambiguity (specific source + imprecise number), and high ambiguity (vague source + imprecise number). Source ambiguity and numerical ambiguity were conceptualized as two message-level ambiguity cues that instantiate ambiguity-based hedging in misinformation statements. Lower source specificity was treated as source ambiguity, whereas lower numerical precision was treated as numerical ambiguity. Participants were randomly assigned to one of the four experimental conditions. Within each condition, each participant evaluated five misinformation items corresponding to that condition. Moreover, because all target claims were debunked misinformation items, the present design assesses participants' rejection of known false claims rather than their ability to distinguish between true and false information.

An a priori power analysis was conducted using G^*^Power (Faul et al., 2007). A one-way ANOVA was used for initial sample-size planning, rather than as a model-specific power estimate for the mixed-effects analyses.With α = 0.05, power = 0.80, and a medium effect size (Cohen's f = 0.25), the minimum required sample size was estimated to be 180 participants. This ANOVA-based estimate should therefore be interpreted as an approximation, particularly for small effects or interaction effects in the mixed-effects models.

Participants were university students majoring in English. All were native speakers of Mandarin Chinese or had received formal education in Mandarin Chinese, and all reported normal or corrected-to-normal vision. Because participants were recruited from English-major programs, passing CET-4 was recorded as part of their academic background rather than as a variable of theoretical interest.

A total of 182 participants were included in this study. Demographic characteristics are presented in Table 1. The four experimental conditions did not differ significantly in age, *F*_(3, 178)_ = 0.579, *p* = 0.629, or gender distribution, χ^2^(3) = 5.671, *p* = 0.129; Fisher's exact test yielded a similar result, *p* = 0.147.

**Table 1:** Participant demographics by condition.

Experimental condition	Source	Numerical	*N*	Age M (SD)	Female *n* (%)
Low ambiguity	Specific	Precise	46	19.89 (2.54)	32 (69.6%)
Source ambiguity	Vague	Precise	46	19.80 (2.34)	38 (82.6%)
Numerical ambiguity	Specific	Imprecise	44	20.02 (2.88)	39 (88.6%)
High ambiguity	Vague	Imprecise	46	19.39 (1.78)	38 (82.6%)
Total	182			19.77 (2.39)	147 (80.8%)

All participants were active social media users (at least 5 h *per* day) and reported no known cognitive impairments. The study was approved by the relevant ethics committee, and all participants provided informed consent prior to participation.

### 2.2 Materials

Experimental stimuli were drawn from piyao.org.cn, a rumor-debunking platform operated by Xinhua News and the Cyberspace Administration of China. Stimuli were selected according to the following criteria: they were published between January 2023 and December 2025, concerned everyday life topics, contained both specific source attributions and precise numerical expressions in their original form, and did not involve sensitive political or religious content. The stimulus set was therefore intended to allow controlled manipulation of source and numerical ambiguity rather than to represent the full range of Chinese misinformation topics. For each baseline item, we created three additional versions by systematically manipulating source ambiguity and numerical ambiguity. In the source-ambiguity version, precise numerical information was retained while specific source attributions were replaced with vague or less identifiable descriptions. This manipulation was guided by prior work on source attribution and source identifiability, in which identified sources are contrasted with anonymous or less identifiable sources (Pjesivac and Rui, 2014). In the numerical-ambiguity version, explicit source attributions were preserved while precise numerical expressions were replaced with imprecise ranges or qualitative quantifiers, consistent with experimental work on vagueness in quantity expressions (Green and van Deemter, 2012). For example, named institutions or traceable publication sources were replaced with generic expressions such as some experts or research shows, whereas exact numerical expressions were replaced with less specific expressions such as several or a significant reduction. The high-ambiguity version combined both manipulations, and the four versions of one stimulus are provided in supplementary materials (https://doi.org/10.5281/zenodo.20842745).

To verify the effectiveness of the manipulation, two reviewers who were blind to the study hypotheses evaluated each stimulus version on two 7-point scales: source specificity (*How specific was the information source?* 1 = very ambiguous, 7 = very clear), and numerical specificity (*How specific was the numerical information?* 1 = very ambiguous, 7 = very clear). Inter-rater reliability was assessed using two-way random-effects intraclass correlation coefficients for absolute agreement. The average-measure ICCs were satisfactory for source specificity, ICC = 0.768, and high for numerical specificity, ICC = 0.928. Ratings were averaged across the two raters for each item-version combination, and paired-samples *t* tests were conducted at the item level. Versions with specific source attributions received significantly higher source-specificity ratings than versions with vague source attributions, *t*_(4)_ = 16.00, *p* < 0.001. Likewise, versions with precise numerical expressions received significantly higher numerical-specificity ratings than versions with imprecise numerical expressions, *t*_(4)_ = 27.10, *p* < 0.001, confirming that the intended ambiguity manipulations were successfully implemented.

Importantly, all materials were factually false because of factual inaccuracies or faulty reasoning rather than ambiguity in wording. We also conducted an additional check to ensure that the core false claim remained unchanged across versions. Eight students who did not participate in the main experiment first read the original version of each statement and then used a 7-point Likert scale (1 = not at all preserved, 7 = completely preserved) to rate the extent to which the core false claim was preserved in the other three versions. All three versions were rated significantly above the scale midpoint (all Ms > 5, all *ps* < 0.01), suggesting that only the degree and type of linguistic ambiguity were manipulated.

In addition, because each item was presented as a short misinformation passage rather than as a single isolated sentence or a long news article. we calculated the character count of each item version and compared character counts across the four versions. The stimuli ranged from 118 to 130 Chinese characters in length, with an average length of approximately 126 characters. A repeated-measures ANOVA with Greenhouse-Geisser correction suggested a possible overall effect of version on character count, *F*_(2.07, 8.28)_ = 4.65, *p* = 0.043. However, Holm-adjusted pairwise comparisons between versions were not significant (all *ps* ≥ 0.245). A non-parametric Friedman test was likewise non-significant, $\chi_{\left(3\right)}$ = 5.00, *p* = 0.172. To further rule out length as a potential confound, we included item-level character count as a covariate in all subsequent mixed-effects analyses.

### 2.3 Procedure

The experiment was conducted online using the Credamo platform (www.credamo.com). No time pressure was imposed during task completion, and participants were instructed to respond according to their own judgment.

After completing the informed consent procedure, participants first provided demographic information, including gender, age, and time spent online. They were then automatically randomized by the Credamo system to one of the four between-subjects conditions. The presentation order of the misinformation stimuli was randomized across participants.

In each trial, participants first made a dichotomous veracity judgment, and reaction time was recorded from stimulus onset to the participant's key-press response. They then rated their sharing intention for the item on a 7-point Likert scale (1 = extremely unlikely, 7 = extremely likely).

After completing the main task, participants rated the materials in terms of comprehensibility (1 = very difficult, 7 = very easy) and perceived clarity (1 = very ambiguous, 7 = very clear) on separate 7-point Likert scales. These measures served as participant-based checks on the perceived comprehensibility and clarity of the materials.

After the experiment, all participants received a full debriefing identifying the materials as verified misinformation and explaining the scientific purpose of the study. Compensation was administered after successful task completion.

### 2.4 Data analysis

Misinformation rejection was measured by whether participants classified each debunked misinformation statement as false. Reaction time (hereafter, RT) was defined as the interval between stimulus onset and the participant's response to the veracity-judgment question.

To ensure data quality, we first removed trials with excessively fast or slow reaction times as part of our data trimming procedure. Specifically, RTs shorter than 200 ms or more than 3 SDs from the overall mean were excluded as outliers. This resulted in the removal of 14 trials, reducing the total number of observations from 910 to 896. As all participants retained at least three valid trials, no participants were excluded from subsequent analyses. Given the substantial positive skewness (2.33) and kurtosis (9.35) of the raw RT distribution, RTs were log-transformed to better approximate normality. The Shapiro–Wilk test indicated a significant deviation from normality prior to transformation, W = 0.756, *p* < 0.001, which improved substantially following transformation (skewness = −0.75, kurtosis = 4.70, W = 0.951, *p* < 0.001).

All analyses were conducted in R (R Core Team, 2025) using mixed-effects models fitted with the *lme4* and *lmerTest* packages (Bates et al., 2015). Source ambiguity and numerical ambiguity were dummy coded as binary predictors (source: 0 = specific, 1 = vague; numerical: 0 = precise, 1 = imprecise) To account for repeated observations nested within participants and the small number of stimulus items, all models included random intercepts for participants and items. Misinformation rejection was analyzed using generalized linear mixed-effects models with a binomial error distribution and *logit link*, whereas sharing intention and log-transformed RTs were analyzed using linear mixed-effects models.

Fixed effects in generalized mixed-effects models were evaluated using Wald z tests, whereas fixed effects in linear mixed-effects models were evaluated using *t* tests with Satterthwaite-approximated denominator degrees of freedom.

Model comparisons were conducted using likelihood-ratio tests based on maximum-likelihood estimation. Results are reported as ORs for the misinformation-rejection models and unstandardized coefficients (*B*) for the linear models. All tests were two-tailed, with α = 0.05.

## 3 Results

### 3.1 Manipulation check

Descriptive statistics for perceived clarity and perceived comprehensibility across conditions are presented in Table 2.

**Table 2:** Descriptive statistics for perceived clarity and comprehensibility across conditions.

Experimental condition	*N*	Perceived clarity M	Perceived clarity SD	Perceived comprehensibility M	Perceived comprehensibility SD
Low ambiguity	46	5.17	1.65	5.72	1.19
Source ambiguity	46	3.74	1.78	5.46	0.91
Numerical ambiguity	44	4.50	1.89	5.64	1.08
High ambiguity	46	3.35	1.83	5.87	0.96

Overall, perceived clarity differed significantly across the four conditions, *F*_(3, 178)_ = 9.463, *p* < 0.001. *Post hoc* Tukey tests showed that the source ambiguity condition was rated significantly lower in clarity than the low ambiguity condition (MD = −1.435, *p* < 0.001), and the high ambiguity condition was also rated significantly lower in clarity than the low ambiguity condition (MD = −1.826, *p* < 0.001). In addition, the high ambiguity condition received significantly lower clarity ratings than the numerical ambiguity condition (MD = −1.152, *p* = 0.014). These findings indicate that the ambiguity manipulation successfully altered participants' perceptions of stimulus clarity.

Furthermore, the overall comprehensibility score was significantly higher than the scale midpoint of 4, *t*_(181)_ = 21.642, *p* < 0.001, M = 5.67, *Cohen's d* = 1.60. More specifically, all four conditions showed comprehensibility ratings significantly above 4 (Holm-adjusted *p*s < 0.001, all Ms > 5.4), suggesting that the manipulation changed perceived clarity without substantially reducing comprehensibility.

### 3.2 Rejection of debunked misinformation

Descriptive statistics by condition are presented in Table 3. Rejection rates for debunked misinformation claims ranged from 55.1% to 77.1% across the four conditions. Mean raw RTs ranged from 16.83s to 22.28s, and mean sharing-intention ratings ranged from 3.02 to 3.33.

**Table 3:** Descriptive statistics across conditions.

Experimental condition	Trials	Rejection rate (%)	Raw RT, M (SD), s	Sharing intention, M (SD)
Low ambiguity	227	55.1	20.86 (19.37)	3.15 (1.39)
Source ambiguity	224	73.7	22.28 (19.60)	3.25 (1.32)
Numerical ambiguity	218	67.4	18.58 (16.42)	3.02 (1.54)
High ambiguity	227	77.1	16.83 (16.26)	3.33 (1.27)

Results of all model comparisons were reported in Table A1. Specifically, maximum-likelihood model comparisons showed that including the interaction between source and numerical ambiguity did not significantly improve model fit relative to the main-effects model, χ^2^_(1)_ = 1.30, *p* = 0.254. The interaction between source ambiguity and numerical ambiguity was not significant, *b* = −0.427, *p* = 0.250, and was therefore not retained in the final model.

As shown in Table 4, both source ambiguity, *b* = 0.817, *p* < 0.001, and numerical ambiguity, *b* = 0.451, *p* = 0.016, significantly increased the odds of rejecting debunked misinformation as false.

**Table 4:** Fixed-effects estimates for the final misinformation-rejection model.

Predictor	*b* (logit)	SE	*z*	*p*	OR	95% CI (OR)
(Intercept)	0.434	0.517	0.839	0.401	1.543	(0.56, 4.25)
Source (vague)	0.817	0.188	4.339	< 0.001	2.263	(1.57, 3.27)
Numerical (imprecise)	0.451	0.187	2.421	0.016	1.571	(1.09, 2.26)

Predicted rejection-rate estimates from the mixed-effects logistic regression model are reported in Table 5. The predicted rejection rate was lowest in the low-ambiguity condition (60.67%) and highest in the high-ambiguity condition (84.58%), with the source-ambiguity (77.74%) and numerical-ambiguity (70.79%) conditions falling in between.

**Table 5:** Predicted rejection rates from the mixed-effects logistic regression model.

Experimental condition	Predicted rejection rate	SE	95% CI
Low ambiguity	60.67%	12.33%	(35.91%, 80.95%)
Source ambiguity	77.74%	9.01%	(55.72%, 90.65%)
Numerical ambiguity	70.79%	10.74%	(46.69%, 87.02%)
High ambiguity	84.58%	6.84%	(66.25%, 93.87%)

### 3.3 Willingness to share misinformation

As shown in Table 6, in the total-effects model, neither source ambiguity, *B* = 0.210, *p* = 0.221, nor numerical ambiguity, *B* = −0.011, *p* = 0.947 significantly predicted sharing intention.

**Table 6:** Linear mixed-effects models predicting post-judgment sharing intention.

Predictor	Total-effects model				Rejection-adjusted model			
	*B*	SE	*t*	*p*	*B*	SE	*t*	*P*
(Intercept)	3.094	0.165	18.727	< 0.001	3.343	0.180	18.601	< 0.001
Source	0.210	0.172	1.227	0.221	0.272	0.168	1.618	0.107
Numerical	−0.011	0.172	−0.067	0.947	0.022	0.168	0.132	0.896
Misinformation rejection	—	—	—	—	−0.435	0.073	−5.989	< 0.001

We then added trial-level misinformation rejection as a predictor. This significantly improved model fit, χ^2^_(1)_ = 34.53, *p* < 0.001. In the rejection-adjusted model, trial-level misinformation rejection was associated with lower sharing intention, *B* = −0.435, *t* = −5.99.

After adjustment for the trial-level misinformation rejection, the residual effect of source ambiguity was modest, *B* = 0.272, *p* = 0.107, whereas numerical ambiguity remained negligible, *B* = 0.022, *p* = 0.896.

### 3.4 Processing speed during misinformation rejection

Model comparison showed that adding the interaction between source ambiguity and numerical ambiguity did not significantly improve model fit relative to the main-effects model, χ^2^_(1)_ = 0.67, *p* = 0.414.

Accordingly, the more parsimonious main-effects model was retained. In this model, source ambiguity did not significantly predict RTs, *B* = −0.021, *p* = 0.839. By contrast, numerical ambiguity significantly predicted shorter RTs, *B* = −0.253, *p* = 0.015.

As a robustness check, the RT analysis was repeated using correct trials only, and the results of both analysis are presented in Table 7. The interaction between source ambiguity and numerical ambiguity again did not significantly improve model fit, χ^2^(1) = 0.61, *p* = 0.434. The pattern of main effects was unchanged. Source ambiguity was not a significant predictor of RTs, *B* = −0.065, *p* = 0.535, whereas numerical ambiguity significantly predicted shorter RTs, *B* = −0.294, *p* = 0.005.

**Table 7:** Linear mixed-effects models predicting logRT.

Predictor	All trials (logRT)				Correct trials only (logRT)			
	*B*	SE	*t*	*p*	*B*	SE	*t*	*p*
(Intercept)	9.677	0.174	55.625	< 0.001	9.732	0.190	51.286	< 0.001
Source	−0.021	0.103	−0.203	0.839	−0.065	0.104	−0.621	0.535
Numerical	−0.253	0.103	−2.455	0.015	−0.294	0.104	−2.818	0.005

As sensitivity analyses, we additionally controlled for within-item variation in material length by including a within-item centered character-count covariate in all models.

As shown in Table 8, across all models, the within-item centered character-count covariate did not significantly improve model fit, including in the misinformation-rejection model, χ^2^_(1)_ = 0.48, *p* = 0.487, the sharing intention model, χ^2^_(1)_ = 2.13, *p* = 0.144, the all-trial RTs model, χ^2^_(1)_ = 1.67, *p* = 0.197, and the correct-trial RTs model, χ^2^_(1)_ = 3.38, *p* = 0.066. In addition, material length was not a significant predictor in any model. For example, in the correct-trial RT model, its effect was not significant, *B* = 0.064, *p* = 0.067.

**Table 8:** Model comparisons for within-item character-count robustness analyses.

Dependent variable	Model	AIC	BIC	Model comparison χ^2^ (df)	Model comparison *p*	character count effect (*B*)	character count effect *p*
MR	Baseline model (source + numerical)	990.1	1,014.1	–	–	–	–
	+ Within-item character count	991.6	1,020.4	0.48 (1)	0.487	0.087	0.484
Sharing intention	Rejection-adjusted model (source + numerical + MRs)	2,620.4	2,654.0	–	–	–	–
	+ Within-item character count	2,620.3	2,658.7	2.13 (1)	0.144	0.061	0.145
Reaction time (log, all trials)	Baseline model (Source + Numerical)	1,943.3	1,972.1	–	–	–	–
	+ Within-item character count	1,943.6	1,977.2	1.67 (1)	0.197	0.038	0.198
Reaction time (log, correct trials only)	Baseline model (source + numerical)	1,324.2	1,350.7	–	–	–	–
	+ Within-item character count	1,322.8	1,353.7	3.38 (1)	0.066	0.064	0.067

Importantly, the overall pattern of results was largely unchanged. Source ambiguity and numerical ambiguity remained significant predictors in the misinformation-rejection model (*b* = 0.822, *p* < 0.001; *b* = 0.565, *p* = 0.022, respectively). In the sharing-intention model, misinformation rejection continued to negatively predict sharing intention (*B* = −0.433, *p* < 0.001), whereas neither ambiguity variable was significant. For RTs, source ambiguity was consistently non-significant in both the all-trial model (*B* = −0.025, p = 0.810) and the correct-trial model (*B* = −0.075, *p* = 0.473), while the effect of numerical ambiguity was marginal in the former (*B* = −0.212, *p* = 0.051) but significant in the latter (*B* = −0.232, *p* = 0.035).

## 4 General discussion

### 4.1 Ambiguity-related cues and misinformation rejection

Our findings suggest that both source ambiguity and numerical ambiguity were associated with a higher likelihood of rejecting misinformation as false. This pattern suggests hedged formulations may have been interpreted as indicating weaker evidential grounding. One possible explanation is that the explicit veracity-judgment task made accuracy salient, thereby encouraging participants to treat vague sources and imprecise numbers as cues to weak evidential support. This interpretation is broadly consistent with research showing that ambiguous cues can reduce perceived trustworthiness and diminish the use of information in decision making (Schneider et al., 2022). It is also compatible with *epistemic vigilance theory*, which proposes that recipients evaluate communicated information by attending to cues of source reliability and content credibility (Sperber et al., 2010). It is further supported by evidence that message-level linguistic features shape credibility assessment and detection performance (Zhao et al., 2022), and this interpretation should be considered alongside evidence that ambiguity can sometimes complicate veracity judgments and increase the demand for disambiguating information (Guigon et al., 2024). At the same time, this finding should not be taken to suggest that ambiguity is generally beneficial for misinformation detection. Prior work has also shown that clear sourcing and precise quantitative information can support truth discernment (van der Bles et al., 2019; Prike et al., 2024). The present findings therefore warrant a more circumscribed interpretation: ambiguity-related cues seem to have signaled limited evidential grounding. This role, however, appears to have been confined to the rejection of debunked misinformation rather than extending across all measured responses. Accordingly, this mechanistic interpretation should be treated as tentative, as the present study measured behavioral outcomes rather than underlying psychological processes.

### 4.2 The limited role of ambiguity-related cues in sharing intention

The sharing intention results help clarify this boundary. While source ambiguity and numerical ambiguity did not predict sharing intention, greater rejection of debunked misinformation was associated with lower sharing intention, suggesting that participants' willingness to share misinformation was more closely related to whether they correctly classified each debunked claim as false. This finding is consistent with the view that misinformation sharing is driven in part by insufficient attention to accuracy, such that people often share content they could correctly evaluate when explicitly asked to assess its accuracy (Pennycook and Rand, 2021; Pennycook et al., 2020; Lutzke et al., 2019).

Importantly, the present effect emerged at the trial level, such that sharing intention increased specifically on trials in which participants misjudged veracity. This suggests that inaccurate evaluation was associated not merely with a general tendency to share misinformation, but with greater willingness to share specific content on trials where veracity was judged incorrectly. The findings are further supported by intervention research indicating that drawing attention to accuracy can reduce misinformation sharing. For example, Pennycook et al. (2021) showed that simple accuracy prompts can decrease the online sharing of misinformation. Nonetheless, this finding should be interpreted with caution, as the sequential judgment procedure may have made participants more attentive to accuracy than they would typically be in real social media contexts.

### 4.3 Numerical ambiguity and the timing of misinformation rejection

At the same time, the RT findings show that the two forms of ambiguity were not equivalent in their temporal profile during judgment. Numerical ambiguity, but not source ambiguity, was associated with faster responses, and this pattern remained when the analysis was restricted to correct trials. This finding suggests that imprecise numerical expressions may have provided a relatively salient cue during veracity evaluation. Reduced numerical specificity may have made the claim appear less certain or less strongly supported at an early stage of judgment, which may in turn have contributed to faster rejection. This interpretation can be understood in light of previous evidence that numerical precision is often taken as a cue to confidence or evidential strength, whereas numerical imprecision may convey weaker precision or evidential support (Jerez-Fernandez et al., 2014; Zhang and Schwarz, 2013).

In contrast, although source ambiguity was associated with higher misinformation rejection, it did not produce a significant RT effect. This divergence between veracity judgments and RTs is consistent with the view that explicit evaluations and response-time measures may capture different aspects of validation and evaluation (Wertgen and Richter, 2020). One possible explanation is that the source manipulation primarily changed the specificity and traceability of the attributed source, thereby shaping credibility evaluation without systematically altering the time required to reach a veracity judgment. This interpretation is also consistent with text-validation research showing that source credibility can modulate validation-related reading times, but that such effects tend to depend on the plausibility of the information rather than appearing as a uniform main effect (Wertgen and Richter, 2020).

Importantly, these effects were not reducible to superficial textual variation. Message length did not significantly predict either rejection of debunked misinformation claims or RTs, suggesting that the observed patterns are more plausibly attributable to message-level cues than to simple differences in item length. Overall, this asymmetry suggests that, although both source ambiguity and numerical ambiguity were associated with higher rejection rates for debunked misinformation claims, they were not associated with identical response patterns.

### 4.4 Source ambiguity and numerical ambiguity as distinct message-level cues

This broader pattern suggests that source ambiguity and numerical ambiguity are better treated as distinct discourse cues than as interchangeable forms of hedging. A similar pattern has been reported by Kerr et al. (2023), who showed that different forms of uncertainty communication can influence audience evaluations in different ways. One possible account is that source ambiguity may reduce the traceability of the source and weaken the accountability structure of the claim, making it less clear who stands behind the information (e.g., Mahr and Csibra, 2021). Numerical ambiguity, by contrast, may reduce quantitative precision and weaken the impression that the claim is communicated with high confidence or precision (Jerez-Fernandez et al., 2014; Zhang and Schwarz, 2013). More broadly, research on uncertainty communication suggests that expressions of uncertainty can shape how audiences evaluate information quality and evidential support (van der Bles et al., 2020). In this sense, both may be understood as forms of ambiguity-related hedging, but they may affect different dimensions of evidential interpretation. This distinction suggests that different forms of ambiguity-related hedging in misinformation discourse may cue different aspects of evidential weakness and may therefore be associated with misinformation rejection in different ways. Nevertheless, these explanations should be viewed as plausible interpretations rather than demonstrated mechanisms.

### 4.5 Implications, limitations, and future research

The present study has both practical and theoretical implications. Practically, the findings suggest that readers may sometimes use salient ambiguity-related hedging as a discourse cue when evaluating misinformation. At the same time, the results do not indicate that ambiguity-related hedging itself directly reduces sharing. Instead, lower sharing intention was associated with greater rejection of debunked misinformation. This suggests that interventions targeting users' accuracy-focused evaluation may be a promising route for reducing intentions to share misinformation. Possible examples include media literacy training, accuracy prompts, fact-checking cues, and interface designs that encourage reflection before sharing. More specifically, the present findings suggest that such efforts may benefit from drawing readers' attention not only to what a message claims, but also to how its evidential basis is linguistically framed. In particular, vague source attribution and imprecise numerical expression may function as different discourse cues in misinformation evaluation, pointing to the practical importance of source traceability and numerical specificity in digital language awareness and critical reading.

At the same time, caution is warranted. Ambiguity-related hedging should not be treated as a simple diagnostic marker of falsehood. Vague expressions can sometimes alert readers to unreliability, but they can also obscure factual weakness without necessarily lowering perceived credibility. Their interpretation is likely to depend on context and on how readers construe verbal uncertainty (Brun and Teigen, 1988). Over-reliance on such cues may therefore be misleading (Dias et al., 2020). For this reason, efforts to counter misinformation should not be limited to promoting skepticism toward ambiguous expression, but should also strengthen broader evaluative capacities, including attention to evidence quality, source traceability, and numerical specificity.

Theoretically, the present study contributes to misinformation research by suggesting that ambiguity-related hedging may not be best treated as a uniform feature. Source ambiguity and numerical ambiguity appear to shape evaluation in related but not identical ways. More broadly, the findings suggest that models of misinformation processing should pay closer attention to the linguistic characteristics of message content and to the possibility that such characteristics may influence both veracity judgment and subsequent communicative intention.

Several limitations should also be acknowledged. First, the stimulus set comprised only five debunked misinformation items, mainly concerning everyday-life topics, which limits the generalizability of the findings across topics, linguistic formulations, and misinformation domains, especially politically or ideologically charged misinformation. Second, because all stimuli were false statements, the present results are most directly relevant to misinformation rejection under controlled conditions rather than to veracity judgment in social media environments, where users encounter mixed true and false claims without knowing their truth status in advance. Third, sharing intention was assessed only after participants had made an explicit truth judgment, which limits the extent to which the sharing findings can be taken to reflect spontaneous or naturalistic sharing behavior. This sequential procedure may have made accuracy concerns more salient, leading participants to align their sharing intentions more closely with their veracity judgments than they might in actual social media contexts. Fourth, the sample consisted predominantly of young female English majors, which restricts the generalizability of the findings to broader populations with different disciplinary backgrounds, media literacy levels, or contextual experience with misinformation. In addition, participants' prior familiarity with or prior belief in the target misinformation was not directly measured. Finally, although epistemic vigilance offers a useful interpretive framework, neither epistemic vigilance nor related processes such as perceived evidential weakness, source traceability, and numerical precision were directly measured. Future research should therefore examine these questions using larger and more diverse samples and stimulus sets, include both true and false materials, directly assess participants' prior familiarity with or belief in the target claims, and test the proposed mechanism more directly.

## 5 Conclusion

Our findings suggest that ambiguity-related hedging does not necessarily make misinformation more persuasive in the present experimental context. Instead, under the conditions tested here, both source ambiguity and numerical ambiguity were associated with greater rejection of debunked misinformation claims. At the same time, these two ambiguity-based cues did not operate identically across outcomes, underscoring the need for a more differentiated account of message-level cues in misinformation evaluation.

These findings contribute to research on language, communication, and misinformation by suggesting that veracity judgment may be shaped not only by propositional content, but also by how evidential support is linguistically framed. More broadly, they suggest that misinformation literacy interventions may benefit from encouraging readers to attend to source traceability, numerical specificity, and other message-level cues of evidential support.

## Figures and Tables

**Table A1:** Model comparisons for misinformation-rejection, sharing intention, and all-trial RT analyses.

Outcome	Comparison	Main-effects/baseline model	Expanded model	χ^2^(df)	*p*	Decision
MR	Test of source × numerical interaction	MRs ~ source + numerical + (1 | id) + (1 | items)	MRs ~ source ^*^ numerical + (1 | id) + (1 | items)	1.30 (1)	0.254	Interaction not retained
Sharing intention	Addition of trial-level misinformation rejection	STs ~ source + numerical + (1 | id) + (1 | items)	STs ~ source + numerical + MRs + (1 | id) + (1 | items)	34.53 (1)	< 0.001	Misinformation rejection retained
All-trial RT (log-transformed)	Test of source × numerical interaction	logRT ~ source + numerical + (1 | id) + (1 | items)	logRT ~ source ^*^ numerical + (1 | id) + (1 | items)	0.67 (1)	0.414	Interaction not retained

## Data Availability

The data supporting this study, including the supplementary materials, have been deposited in Zenodo and are available at: https://doi.org/10.5281/zenodo.20842745.

## References

[B1] Bates D. Mächler M. Bolker B. Walker S. (2015). Fitting linear mixed-effects models using lme4. J. Stat. Softw. 67, 1–48. doi: 10.18637/jss.v067.i01

[B2] Brun W. Teigen K. H. (1988). Verbal probabilities: ambiguous, context-dependent, or both? Organ. Behav. Hum. Decis. Process. 41, 390–404. doi: 10.1016/0749-5978(88)90036-2

[B3] Budescu D. V. Wallsten T. S. (1995). “Processing linguistic probabilities: general principles and empirical evidence,” in Psychology of Learning and Motivation, Vol. 32 (San Diego, CA: Academic Press), 275–318.

[B4] Collins R. N. Mandel D. R. (2019). Cultivating credibility with probability words and numbers. Judgm. Decis. Mak. 14, 683–695. doi: 10.1017/S1930297500005404

[B5] Dias N. Pennycook G. Rand D. G. (2020). Emphasizing publishers does not effectively reduce susceptibility to misinformation on social media. HKS Misinform. Rev. 1, 1–12. doi: 10.37016/mr-2020-001

[B6] El Mikati I. K. Hoteit R. Harb T. El Zein O. Piggott T. Melki J. . (2023). Defining misinformation and related terms in health-related literature: scoping review. J. Med. Internet Res. 25:e45731. doi: 10.2196/45731 37556184 PMC10414029

[B7] Faul F. Erdfelder E. Lang A.-G. Buchner A. (2007). G^*^Power 3: a flexible statistical power analysis program for the social, behavioral, and biomedical sciences. Behav. Res. Methods 39, 175–191. doi: 10.3758/BF03193146 17695343

[B8] Fu C. Zhang J. Kang X. (2024). True or false? Linguistic and demographic factors influence veracity judgment of COVID-19 rumors. Humanit. Soc. Sci. Commun. 11:410. doi: 10.1057/s41599-024-02935-4

[B9] Green M. J. van Deemter K. (2012). “Vagueness in referring expressions of quantity: effects on the audience,” in SIPI 2012 Proceedings (Aberdeen).

[B10] Guigon V. Villeval M. C. Dreher J.-C. (2024). Metacognition biases information seeking in assessing ambiguous news. Commun. Psychol. 2:122. doi: 10.1038/s44271-024-00170-w 39702410 PMC11659316

[B11] Hyland K. (1998). Hedging in Scientific Research Articles. Amsterdam: John Benjamins.

[B12] Jensen J. D. (2008). Scientific uncertainty in news coverage of cancer research: effects of hedging on scientists' and journalists' credibility. Hum. Commun. Res. 34, 347–369. doi: 10.1111/j.1468-2958.2008.00324.x

[B13] Jerez-Fernandez A. Angulo A. N. Oppenheimer D. M. (2014). Show me the numbers: precision as a cue to others' confidence. Psychol. Sci. 25, 633–635. doi: 10.1177/0956797613504301 24317423

[B14] Kerr J. van der Bles A.-M. Dryhurst S. Schneider C. R. Chopurian V. Freeman A. L. J. . (2023). The effects of communicating uncertainty around statistics, on public trust. R. Soc. Open Sci. 10:230604. doi: 10.1098/rsos.230604 38026007 PMC10663791

[B15] Lakoff G. (1972). “Hedges: a study in meaning criteria and the logic of fuzzy concepts,” in Papers from the Eighth Regional Meeting of the Chicago Linguistic Society (Chicago, IL), 183–228.

[B16] Lazer D. M. J. Baum M. A. Benkler Y. Berinsky A. J. Greenhill K. M. Menczer F. . (2018). The science of fake news. Science 359, 1094–1096. doi: 10.1126/science.aao2998 29590025

[B17] Lewandowsky S. Ecker U. K. H. Seifert C. M. Schwarz N. Cook J. (2012). Misinformation and its correction: continued influence and successful debiasing. Psychol. Sci. Public Interest 13, 106–131. doi: 10.1177/1529100612451018 26173286

[B18] Luo M. Hancock J. T. Markowitz D. M. (2022). Credibility perceptions and detection accuracy of fake news headlines on social media: effects of truth-bias and endorsement cues. Commun. Res. 49, 171–195. doi: 10.1177/0093650220921321

[B19] Lutz B. Adam M. Feuerriegel S. Pröllochs N. Neumann D. (2024). Which linguistic cues make people fall for fake news? A comparison of cognitive and affective processing. Proc. ACM Hum.-Comput. Interact. 8, 1–22. doi: 10.1145/364103039286336

[B20] Lutzke L. Drummond C. Slovic P. Árvai J. (2019). Priming critical thinking: simple interventions limit the influence of fake news about climate change on Facebook. Glob. Environ. Change 58:101964. doi: 10.1016/j.gloenvcha.2019.101964

[B21] Mahr J. B. Csibra G. (2021). The effect of source claims on statement believability and speaker accountability. Mem. Cognit. 49, 1505–1525. doi: 10.3758/s13421-021-01186-x PMC8563530 34128185

[B22] Mitra T. Wright G. P. Gilbert E. (2017). “A parsimonious language model of social media credibility across disparate events,” in Proceedings of the ACM Conference on Computer-Supported Cooperative Work and Social Computing (CSCW '17) (New York, NY: ACM), 126–145.

[B23] Muhammed S. T. Mathew S. K. (2022). The disaster of misinformation: a review of research in social media. Int. J. Data Sci. Anal. 13, 271–285. doi: 10.1007/s41060-022-00311-6 35194559 PMC8853081

[B24] Orr S. Kronmüller E. Shetreet E. (2026). Hedging the truth: the evaluation of truth under deceptive (un)certainty. J. Pragmat. 259, 86–100. doi: 10.1016/j.pragma.2026.03.016

[B25] Pennycook G. Epstein Z. Mosleh M. Arechar A. A. Eckles D. Rand D. G. (2021). Shifting attention to accuracy can reduce misinformation online. Nature 592, 590–595. doi: 10.1038/s41586-021-03344-2 33731933

[B26] Pennycook G. McPhetres J. Zhang Y. Lu J. G. Rand D. G. (2020). Fighting COVID-19 misinformation on social media: experimental evidence for a scalable accuracy-nudge intervention. Psychol. Sci. 31, 770–780. doi: 10.1177/0956797620939054 32603243 PMC7366427

[B27] Pennycook G. Rand D. G. (2019). Lazy, not biased: susceptibility to partisan fake news is better explained by lack of reasoning than by motivated reasoning. Cognition 188, 39–50. doi: 10.1016/j.cognition.2018.06.011 29935897

[B28] Pennycook G. Rand D. G. (2021). The psychology of fake news. Trends Cogn. Sci. 25, 388–402. doi: 10.1016/j.tics.2021.02.007 33736957

[B29] Pjesivac I. Rui R. (2014). Anonymous sources hurt credibility of news stories across cultures: a comparative experiment in America and China. Int. Commun. Gaz. 76, 641–660. doi: 10.1177/1748048514548534

[B30] Prike T. Butler L. H. Ecker U. K. H. (2024). Source-credibility information and social norms improve truth discernment and reduce engagement with misinformation online. Sci. Rep. 14:6900. doi: 10.1038/s41598-024-57560-7 38519569 PMC10960008

[B31] R Core Team (2025). R: A Language and Environment for Statistical Computing. Vienna: R Foundation for Statistical Computing.

[B32] Rapp D. N. (2008). How do readers handle incorrect information during reading? Mem. Cognit. 36, 688–701. doi: 10.3758/MC.36.3.688 18491506

[B33] Rashkin H. Choi E. Jang J. Y. Volkova S. Choi Y. (2017). “Truth of varying shades: analyzing language in fake news and political fact-checking,” in Proceedings of EMNLP 2017 (Copenhagen: Association for Computational Linguistics), 2931–2937.

[B34] Schneider C. R. Freeman A. L. J. Spiegelhalter D. van der Linden S. (2022). The effects of communicating scientific uncertainty on trust and decision making in a public health context. Judgm. Decis. Mak. 17, 849–882. doi: 10.1017/S1930297500008962

[B35] Solt S. (2011). “Vagueness in quantity: two case studies from a linguistic perspective,” in Understanding Vagueness: Logical, Philosophical and Linguistic Perspectives, eds. P. Cintula, C. G. Fermüller, L. Godo, and P. Hájek (London: College Publication), 157–174.

[B36] Sperber D. Clément F. Heintz C. Mascaro O. Mercier H. Origgi G. . (2010). Epistemic vigilance. Mind Lang. 25, 359–393. doi: 10.1111/j.1468-0017.2010.01394.x

[B37] Stavrova O. Kleinberg B. Evans A. M. Ivanović M. (2024). Expressions of uncertainty in online science communication hinder information diffusion. PNAS Nexus 3:pgae439. doi: 10.1093/pnasnexus/pgae439 39430220 PMC11489878

[B38] van der Bles A. M. van der Linden S. Freeman A. L. J. Spiegelhalter D. J. (2019). Communicating uncertainty about facts, numbers and science. R. Soc. Open Sci. 6:181870. doi: 10.1098/rsos.181870 31218028 PMC6549952

[B39] van der Bles A. M. van der Linden S. Freeman A. L. J. Spiegelhalter D. J. (2020). The effects of communicating uncertainty on public trust in facts and numbers. Proc. Natl. Acad. Sci. U.S.A. 117, 7672–7678. doi: 10.1073/pnas.1913678117 32205438 PMC7149229

[B40] van Eck C. W. Hameleers M. van der Goot E. (2026). Identifying fake experts: a conceptual framework and case study of illegitimate expertise in climate change and COVID-19 misinformation and its implications for communication theory. Commun. Theory 36, 36–45. doi: 10.1093/ct/qtaf033

[B41] Vosoughi S. Roy D. Aral S. (2018). The spread of true and false news online. Science 359, 1146–1151. doi: 10.1126/science.aap9559 29590045

[B42] Wertgen A. G. Richter T. (2020). Source credibility modulates the validation of implausible information. Mem. Cognit. 48, 1352–1364. doi: 10.3758/s13421-020-01067-9 PMC7683457 32651943

[B43] Zeng H.-K. Wu T.-Y. Atkin D. J. (2023). Check the report and comments: the veracity assessment of unfamiliar news on social media. Digit. Journal. 11, 161–180. doi: 10.1080/21670811.2022.2079541

[B44] Zhang Y. C. Schwarz N. (2013). The power of precise numbers: a conversational logic analysis. J. Exp. Soc. Psychol. 49, 944–946. doi: 10.1016/j.jesp.2013.04.002

[B45] Zhao J. Fu C. (2024). Linguistic indicators for predicting the veracity of online health rumors. Front. Public Health 11:1278503. doi: 10.3389/fpubh.2023.1278503 38269391 PMC10806107

[B46] Zhao J. Fu C. Kang X. (2022). Content characteristics predict the putative authenticity of COVID-19 rumors. Front. Public Health 10:920103. doi: 10.3389/fpubh.2022.920103 36033743 PMC9399738

